# Improvement in oxygenation with high-frequency oscillatory ventilation combined with tracheal gas insufflation is correlated to the extravascular lung water index

**DOI:** 10.1186/cc12059

**Published:** 2013-03-19

**Authors:** CS Vrettou, S Malachias, SG Zakynthinos, SD Mentzelopoulos

**Affiliations:** 1 Evaggelismos General Hospital, Athens, Greece

## Introduction

High-frequency oscillatory ventilation combined with tracheal gas insufflation (HFO-TGI) can significantly improve oxygenation in patients with ARDS. It has been demonstrated that oxygenation in patients with ARDS has a better response to HFO when extravascular lung water is >15 ml/kg body weight (BW). Our aim is to examine whether the extravascular lung water index (ELWI) correlates with changes in PaO_2_/FiO_2 _in patients ventilated with HFO-TGI.

## Methods

Data from 18 sessions of HFO-TGI in six patients were included in the analysis. HFO frequency, oscillatory pressure amplitude, and bias flow were 3.5 Hz, 85 to 95 cmH_2_O, and 40 l/minute, respectively; a 2.5 to 3.5 cmH_2_O tracheal tube cuff leak was used. HFO mean airway pressure (mPaw) exceeded preceding conventional ventilation (CV)-mPaw by 7 to 13 cmH_2_O. PaO_2_/FiO_2_, lung mechanics, and hemodynamics were documented during lung-protective CV (baseline) and 1 hour following the initiation of HFO-TGI ventilation. PULSION PICCOplus v7.0 was used for hemodynamic measurements including the ELWI.

## Results

**Figure 1:**
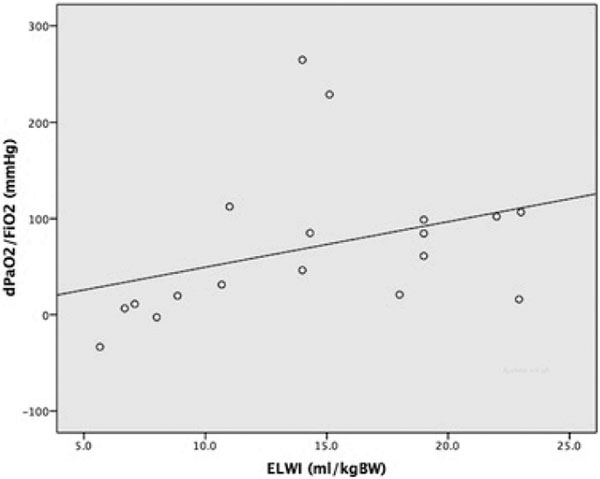
**Scatter plot and fit line of ELWI versus dPaO_2_/FiO_2_**.

Oxygenation (PaO_2_/FiO_2_) improved significantly with HFO-TGI compared with CMV (125.5 ± 54.7 vs. 195.6 ± 108.7, *P <*0.001). Changes in PaO_2_/FiO_2 _were positively correlated with ELWI at baseline (Spearman's ρ = 0.56, *P *= 0.016). See Figure 1. There were no significant changes in patients' fluid balance and hemodynamics including the ELWI.

## Conclusion

Estimation of the ELWI can help to predict the oxygenation response of ARDS patients considered for HFO-TGI ventilation. The possibility that HFO-TGI exerts an effect on pulmonary oedema needs further investigation.
